# Long-term outcomes of 307 patients after complete thymoma resection

**DOI:** 10.1186/s40880-017-0213-8

**Published:** 2017-05-15

**Authors:** Zu-Yang Yuan, Shu-Geng Gao, Ju-Wei Mu, Qi Xue, You-Sheng Mao, Da-Li Wang, Jun Zhao, Yu-Shun Gao, Jin-Feng Huang, Jie He

**Affiliations:** Department of Thoracic Surgery, National Cancer Center/Cancer Hospital, Chinese Academy of Medical Sciences and Peking Union Medical College, Beijing, 100021 P. R. China

**Keywords:** Thymoma, Complete resection, Recurrent thymoma, Prognosis

## Abstract

**Background:**

Thymoma is an uncommon tumor without a widely accepted standard care to date. We aimed to investigate the clinicopathologic variables of patients with thymoma and identify possible predictors of survival and recurrence after initial resection.

**Methods:**

We retrospectively selected 307 patients with thymoma who underwent complete resection at the Cancer Hospital, Chinese Academy of Medical Sciences and Peking Union Medical College (Beijing, China) between January 2003 and December 2014. The associations of patients’ clinical characteristics with prognosis were estimated using Cox regression and Kaplan–Meier survival analyses.

**Results:**

During follow-up (median, 86 months; range, 24–160 months), the 5- and 10-year disease-free survival (DFS) rates were 84.0% and 73.0%, respectively, and the 5- and 10-year overall survival (OS) rates were 91.0% and 74.0%, respectively. Masaoka stage (*P* < 0.001), World Health Organization (WHO) histological classification (*P* < 0.001), and postoperative radiotherapy after initial resection (*P* = 0.006) were associated with recurrence (52/307, 16.9%). Multivariate analysis revealed that, after initial resection, WHO histological classification and Masaoka stage were independent predictors of DFS and OS. The pleura (25/52, 48.0%) were the most common site of recurrence, and locoregional recurrence (41/52, 79.0%) was the most common recurrence pattern. The recurrence pattern was an independent predictor of post-recurrence survival. Patients with recurrent thymoma who underwent repeated resection had increased post-recurrence survival rates compared with those who underwent therapies other than surgery (*P* = 0.017).

**Conclusions:**

Masaoka stage and WHO histological classification were independent prognostic factors of thymoma after initial complete resection. The recurrence pattern was an independent predictor of post-recurrence survival. Locoregional recurrence and repeated resection of the recurrent tumor were associated with favorable prognosis.

## Background

Thymic epithelial tumors, including thymoma and thymic carcinoma, are the most common tumors in the anterior mediastinum [1], with an incidence of 0.17 cases/100,000 annually in China [2]. Thymoma presents with an indolent course and a favorable prognosis. The 5-year overall survival (OS) rate of patients with thymoma is approximately 90.0% [3–5]. Complete resection is the gold-standard treatment of operable thymoma; radiotherapy and chemotherapy appear to benefit patients with inoperable or incompletely resected tumors [3–5].

Unfortunately, although indolent, the recurrence of thymoma is not infrequent after complete resection. Long-term follow-up is therefore required to determine recurrence rates of thymoma patients who undergo complete resection. The possibility of recurrence associates with disease stage upon diagnosis. The average recurrence rates of primary thymoma after resection are as follows: 4.0%, stage I; 14.0%, stage II; 26.0%, stage III; and 46.0%, stage IV [6]. Recurrence is generally confined to the intrathoracic regions, including locoregional recurrence and intrathoracic dissemination; distant metastases are rare.

Owing to the rarity and indolent nature of thymoma, there is limited and inconsistent information related to patients’ long-term outcomes and possible prognostic factors, particularly those associated with recurrent thymoma. The objective of our study was to analyze the long-term outcomes of a large number of patients with thymoma who underwent complete resection. For this purpose, we evaluated clinicopathologic characteristics of the patients with primary thymoma, investigated the types of treatment of recurrent thymoma after initial resection, and estimated independent predictors of disease-free survival (DFS) and OS.

## Methods

### Patient characteristics

We conducted a retrospective analysis of the medical records of 316 consecutive patients with pathologically confirmed primary thymoma who underwent complete resection in the Department of Thoracic Surgery, Cancer Hospital, Chinese Academy of Medical Sciences and Peking Union Medical College (Beijing, China) between January 2003 and December 2014. The patients undergoing preoperative radiotherapy or chemotherapy were excluded; the patients with primary thymoma initially diagnosed using preoperative computed tomography and confirmed via postoperative pathologic examination were eligible for the present study. The pathologic stage was determined according to the Masaoka staging system, and pathologic type was determined according to the World Health Organization (WHO) histological classification. According to the definitions and policies of the International Thymic Malignancy Interest Group, recurrence is considered when there is strong clinical suspicion without a specific requirement of pathologic proof [7]. The time of recurrence was recorded as the time when a strong clinical suspicion was first aroused [8]. Thus, recurrence was diagnosed according to symptoms and imaging findings. Furthermore, biopsy was not mandatory to confirm recurrent thymoma.

Patients’ demographic and clinicopathologic characteristics were acquired through a review of their medical records. The study followed the guidelines of the Declaration of Helsinki and was approved by the Ethics Committee of the Cancer Hospital, Chinese Academy of Medical Sciences and Peking Union Medical College. Informed consent was not required for this retrospective study.

### Treatments

The thymoma patients with myasthenia gravis (MG) mainly complained of ptosis or weakness, and therefore they did not require special treatment before surgery. Surgical techniques, including video-assisted thoracoscopic surgery (VATS) and transsternal and transthoracic complete resection of primary thymoma, were described in our previous study [9]. All procedures followed the criteria for complete resection. Patients with thymoma who were considered at high risk of recurrence according to intraoperative findings and those with invasive lesions and high-grade malignancy according to postoperative pathologic proof were offered adjuvant radiotherapy. Based on the extent of recurrence, the treatments of recurrent thymoma varied and included surgery combined with adjuvant therapy and nonsurgical treatment (radiotherapy, chemotherapy, or both). Incomplete resection for recurrent thymoma included partial tumor removal and debulking surgery in patients with aggressive or multiple lesions that could not be completely removed. The clinical target volume for postoperative radiotherapy encompassed the entire thymus, surgical region, and potential sites at high risk of recurrence. Among the patients with recurrent thymoma, those undergoing complete resection received irradiation with a definitive dose of 45 to 50 Gy after surgery; the patients with postoperative residual disease and unresectable lesions received irradiation with a target dose of 60 Gy. Cisplatin/doxorubicin-based chemotherapy was administered to patients with recurrent thymoma.

The recurrence patterns included locoregional (mediastinal, diaphragmatic, and pleuropulmonary) and distant (pleural or intraparenchymal pulmonary dissemination) patterns. OS was defined as the interval between the date of surgery and the date of death of any cause or the last follow-up. DFS was defined as the interval between the date of surgery and the first recurrence or the last follow-up. Post-recurrence survival (PRS) was defined as the time of recurrence to death or the last follow-up.

### Statistical analysis

Values of continuous variables are presented as the mean ± standard deviation or the median and range. Categorical variables are presented as numbers of patients and percentages. Patients who were alive at the end of follow-up were censored from the analysis of OS. Cox proportional regression analysis was used to determine the significance of the associations of each variable with OS, DFS, and PRS. Variables associated with survival that were identified in univariate analysis were further tested using a multivariate model. Multivariate analysis was performed using the Cox stepwise proportional-hazards method. The association between Masaoka stage and WHO histological classification and the association between each categorical variable and recurrence rate were evaluated using the Chi square test. Kaplan–Meier method was used to estimate survival stratified according to significant clinical variables, and the log-rank test was used to evaluate the statistical significance of the differences. All tests were two-sided, and *P* < 0.05 indicates statistical significance. All data were analyzed using SPSS 19.0 (IBM, Armonk, NY, USA).

## Results

### Primary thymoma

Of the 316 patients, nine were excluded because of preoperative radiotherapy or chemotherapy, and 307 were included in this study. The demographic and clinical characteristics of the 307 patients with thymoma are shown in Table 1. These patients aged 50.5 years (mean), with primary tumors of 6.5 cm (mean) in diameter. The median follow-up was 86 months (range, 24–160 months). The WHO histological classification was highly associated with Masaoka stage (*P* < 0.001). Postoperative radiotherapy was significantly associated with WHO histological classification (*P* < 0.001) and Masaoka stage (*P* < 0.001). Postoperatively, of 58 patients with MG symptoms, 37 (63.8%) experienced remission of MG-related symptoms, 16 (27.6%) did not improve, and 5 (8.6%) suffered MG crises. The five patients who suffered MG crises required mechanical ventilation and were administered intravenous pyridostigmine bromide.

**Table 1:** Characteristics of 307 patients with primary thymoma and their association with recurrence

Variable	Whole cohort (*n* = 307)	Patients with recurrence (*n* = 52)^a^	*P*
Age (years)			0.314
≤60	244 (79.5)	44 (18.0)	
>60	63 (20.5)	8 (12.7)	
Sex			0.942
Female	149 (48.5)	25 (16.8)	
Male	158 (51.5)	27 (17.1)	
Tumor size (cm)			0.950
≤6.5	170 (55.4)	29 (17.1)	
>6.5	137 (44.6)	23 (16.8)	
Myasthenia gravis			0.749
Yes	58 (18.9)	9 (15.5)	
No	249 (81.1)	43 (17.3)	
WHO histological classification			<0.001
A	19 (6.1)	1 (5.3)	
AB	99 (32.2)	5 (5.1)	
B1	61 (19.9)	7 (11.5)	
B2	83 (27.0)	26 (31.3)	
B3	45 (14.7)	13 (28.9)	
Masaoka stage			<0.001
I	74 (24.1)	3 (4.1)	
II	170 (55.4)	17 (10.0)	
III	52 (16.9)	24 (46.2)	
IVa	11 (3.6)	8 (72.7)	
Surgical approach			0.590
Transsternal	97 (31.6)	14 (14.4)	
Transthoracic	140 (45.6)	27 (19.3)	
VATS	70 (22.8)	11 (15.7)	
Postoperative radiotherapy			0.006
Yes	142 (46.3)	15 (10.6)	
No	165 (53.7)	37 (22.4)	

All values are presented as number of patients followed by percentage in parentheses

*WHO* World Health Organization, *VATS* video-assisted thoracoscopic surgery

^a^The percentages in this column was calculated according to the following formula: the number of patients with recurrence/the number of relevant patients in the whole cohort

### DFS rates

The 5- and 10-year DFS rates were 84.0% and 73.0%, respectively. The median time to recurrence was 71 months (range, 4–160 months). Disease recurrence rate was 16.9% (52/307) after initial complete resection of thymoma, which was associated with the initial Masaoka stage (*P* < 0.001). Administration of adjuvant radiotherapy after complete resection was associated with decreased recurrence rate (*P* = 0.006) (Table 1). Cox univariate regression analysis revealed that the following variables were significantly associated with DFS: (1) WHO histological classification (type B2 and type B3 vs. type A + B, all *P* < 0.001); (2) Masaoka stage (stage III and stage IVa vs. stage I, all *P* < 0.001); and (3) postoperative radiotherapy (yes vs. no, *P* = 0.010). Cox multivariate regression analysis identified the independent prognostic factors as follows: WHO histological classification (type B2 vs. type A + B, *P* = 0.036) and Masaoka stage (stage III vs. stage I, *P* = 0.020; stage IVa vs. stage I, *P* < 0.001) (Table 2).

**Table 2:** Univariate and multivariate Cox regression analyses of disease-free survival of 307 patients with primary thymoma

Variable	Univariate			Multivariate		
	HR	95% CI	*P* value	HR	95% CI	*P* value
Age (years)						
≤60						
>60	0.733	0.345–1.558	0.420			
Sex						
Female						
Male	1.175	0.680–2.028	0.563			
Tumor size (cm)	1.017	0.913–1.132	0.762			
Myasthenia gravis						
Yes						
No	1.254	0.610–2.580	0.538			
WHO histological classification						
A + AB						
B1	2.513	0.844–7.481	0.098	1.530	0.492–4.759	0.463
B2	6.831	2.810–16.604	<0.001	2.855	1.070–7.621	0.036
B3	7.999	3.032–21.108	<0.001	2.404	0.809–7.141	0.114
Masaoka stage						
I						
II	2.284	0.669–7.801	0.188	1.211	0.326–4.501	0.775
III	12.624	3.800–41.943	<0.001	4.935	1.289–18.894	0.020
IVa	26.872	7.102–101.680	<0.001	13.809	3.230–59.043	<0.001
Surgical approach						
Transthoracic						
Transsternal	0.803	0.421–1.532	0.506			
VATS	1.304	0.635–2.676	0.470			
Postoperative radiotherapy						
Yes						
No	2.212	1.214–4.032	0.010	1.887	0.932–3.823	0.078

*CI* confidence interval,* HR* hazard ratio,* WHO* World Health Organization,* VATS* video-assisted thoracoscopic surgery

**Fig. 1:**
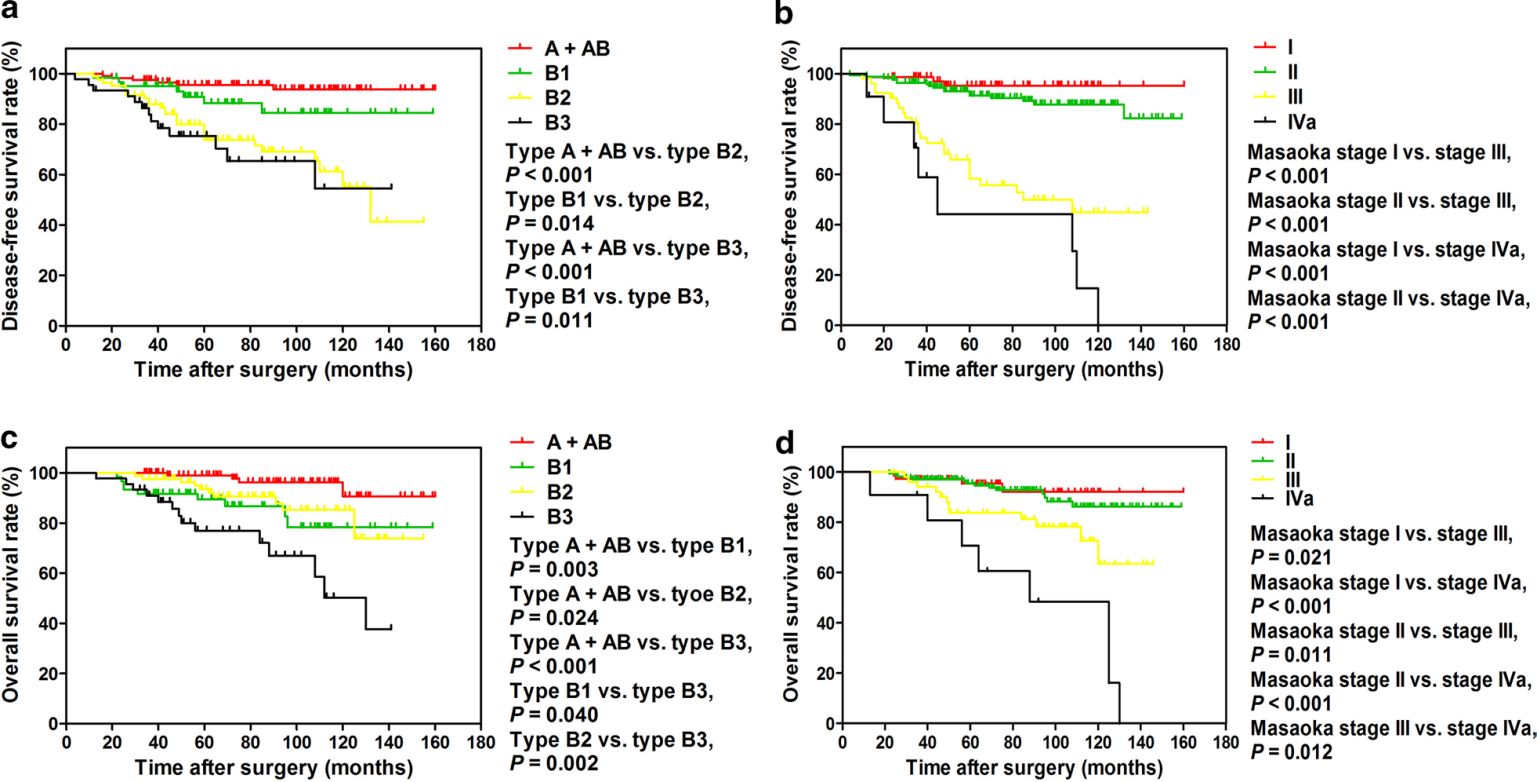
Kaplan–Meier analysis of survival curves for patients with thymoma after initial resection. **a** Disease-free survival curves stratified according to the WHO histological classification. **b** Disease-free survival curves stratified according to the Masaoka stage. **c** Overall survival curves stratified according to the WHO histological classification. **d** Overall survival curves stratified according to the Masaoka stage

Kaplan–Meier analysis and log-rank tests showed that DFS rates were higher in patients with WHO type A + AB and type B1 thymoma than in those with type B2 thymoma (*P* < 0.001 and *P* = 0.014, respectively) and type B3 thymoma (*P* < 0.001 and *P* = 0.011, respectively) (Fig. 1a), whereas the DFS rates were similar between type A + AB and type B1 groups (*P* = 0.087) and between type B2 and type B3 groups (*P* = 0.614). Furthermore, the DFS rates were higher in patients with Masaoka stages I and II thymoma than in patients with stage III thymoma (*P* < 0.001 and *P* < 0.001, respectively) and stage IVa thymoma (*P* < 0.001 and *P* < 0.001, respectively) (Fig. 1b), but were similar between stages I and II groups (*P* = 0.179) as well as between stages III and IVa groups (*P* = 0.079).

### OS rates

Thirty-eight patients (12.4%) died during follow-up. The Cox univariate regression model revealed that the following variables were significantly associated with OS: age (≤60 vs. >60 years, *P* = 0.016), Masaoka stage (stage III vs. stage I, *P* = 0.032; stage IVa vs. stage I, *P* < 0.001), and WHO histological classification (type B1, type B2, and type B3 vs. type A + B, *P* = 0.008, *P* = 0.028, and *P* < 0.001, respectively). Subsequent analysis using the Cox multivariate model revealed the following independent predictors of OS: Masaoka stage (stage IVa vs. stage I, *P* = 0.048) and WHO histological classification (type B1 and type B3 vs. type A + B, *P* = 0.016 and *P* = 0.004, respectively) (Table 3).

**Table 3:** Univariate and multivariate Cox regression analyses of overall survival of 307 patients with primary thymoma

Variable	Univariate			Multivariate		
	HR	95% CI	*P* value	HR	95% CI	*P* value
Age (years)						
≤60						
>60	2.290	1.171–4.481	0.016	1.801	0.850–3.818	0.125
Sex						
Female						
Male	1.187	0.627–2.245	0.599			
Tumor size (cm)	1.114	0.989–1.255	0.075			
Myasthenia gravis						
Yes						
No	1.554	0.647–3.730	0.324			
WHO histological classification						
A + AB						
B1	4.904	1.510–15.930	0.008	4.455	1.329–14.936	0.016
B2	3.605	1.147−11.333	0.028	2.860	0.823–9.940	0.098
B3	11.819	3.884–35.964	<0.001	6.372	1.837–22.103	0.004
Masaoka stage						
I						
II	1.272	0.418–3.877	0.672	0.930	0.291–2.966	0.902
III	3.468	1.116–10.777	0.032	1.812	0.531–6.184	0.342
IVa	11.705	3.495–39.203	<0.001	4.215	1.010–17.593	0.048
Surgical approach						
Transthoracic						
Transsternal	1.001	0.485–2.063	0.998			
VATS	1.455	0.597–3.547	0.409			
Postoperative radiotherapy						
Yes						
No	1.516	0.775–2.966	0.224			

*CI* confidence interval,* HR* hazard ratio,* WHO* World Health Organization,* VATS* video-assisted thoracoscopic surgery

Kaplan–Meier analysis revealed that 5- and 10-year OS rates were 91.0% and 74.0%, respectively. Log-rank test revealed that the patients older than 60 years had significantly a lower OS rate compared with those ≤60 years in age (*P* = 0.013). The OS rate was significantly higher in patients with type A + AB disease than in those with type B1 (*P* = 0.003), type B2 (*P* = 0.024), and type B3 diseases (*P* < 0.001); the patients with type B1 (*P* = 0.040) and type B2 (*P* = 0.002) diseases had higher OS rates than those with type B3 disease; however, the OS rates were similar between patients with types B1 and B2 diseases (*P* = 0.528) (Fig. 1c). Moreover, the OS rate was significantly higher in patients with Masaoka stage I thymoma than in patients with stages III (*P* = 0.021) and IVa thymoma (*P* < 0.001), was significantly higher in patients with stage II disease than in those with stages III (*P* = 0.011) and IVa diseases (*P* < 0.001), and was significantly higher in patients with stage III disease than in those with stage IVa disease (*P* = 0.012); however, the OS rates were similar between the patients with Masaoka stages I and II diseases (*P* = 0.572) (Fig. 1d).

### Recurrent thymoma

**Fig. 2:**
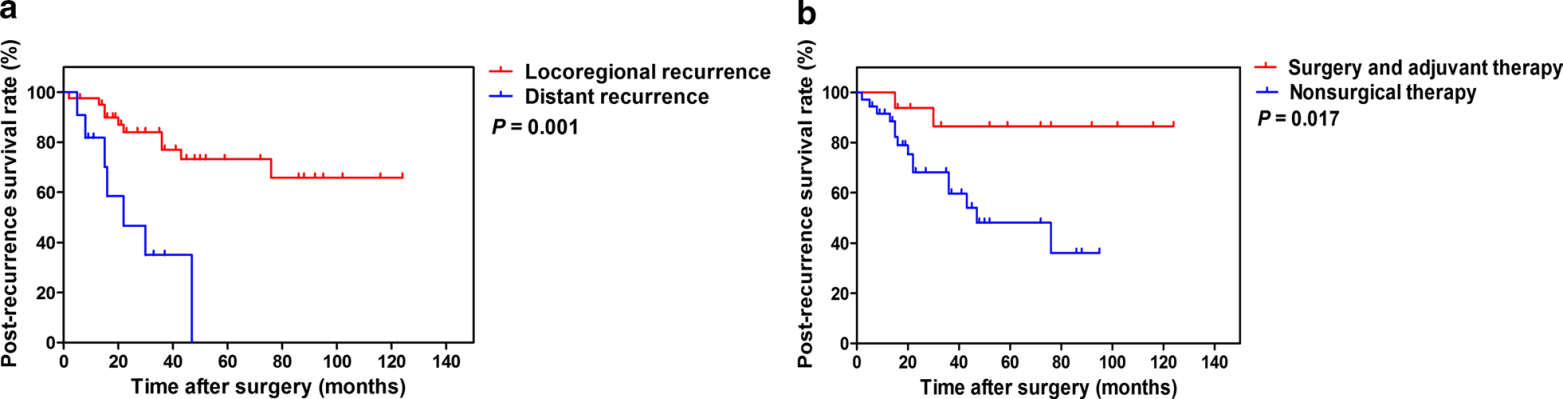
Kaplan–Meier analysis of post-recurrence survival curves for patients with recurrent thymoma. **a** Post-recurrence survival curves stratified according to the recurrence pattern. **b** Post-recurrence survival curves stratified according to the type of therapy

For the 52 patients with recurrent thymoma, recurrence was found in the pleura (25/52, 48.1%), mediastinum (14/52, 26.9%), lung (12/52, 23.1%), and diaphragm (1/52, 1.9%); 41 (78.8%) had locoregional recurrence, and 11 (21.2%) had distant recurrence. Sixteen patients (30.8%) underwent surgical resection combined with adjuvant therapy: 13 patients with locoregional recurrences underwent complete resection plus radiotherapy, and three patients with distant recurrences underwent debulking surgery plus radiochemotherapy. Thirty-six (69.2%) patients did not undergo surgery: 20 patients with locoregional recurrences and two patients with distant recurrences received radiotherapy; four patients with locoregional recurrences and four patients with distant recurrences received chemotherapy; and four patients with locoregional recurrences and two patients with distant recurrences received radiochemotherapy. The median PRS was 35.5 months (range, 2–124 months). Univariate analysis results showed that the following variables were significantly associated with PRS: age (HR = 0.196, 95% CI 0.071–0.541, *P* = 0.002), recurrence pattern (HR = 5.100, 95% CI 1.847–14.078, *P* = 0.002), and treatment type for recurrent tumors (HR = 5.056, 95% CI 1.145–22.324, *P* = 0.032). Multivariate analysis results showed that recurrence pattern was an independent predictive factor for PRS (*P* = 0.003) (Table 4). Kaplan–Meier analysis results showed that longer PRS was significant associated with locoregional recurrence (*P* = 0.001, log-rank test) (Fig. 2a) and surgical resection combined with adjuvant therapy (*P* = 0.017, log-rank test) (Fig. 2b).

**Table 4:** Multivariate Cox regression analysis of post-recurrence survival of 52 patients with recurrent thymoma

Variable	HR	95% CI	*P* value
Age (years)			
≤60			
>60	2.507	0.908–6.921	0.076
Recurrence pattern			
Locoregional recurrence			
Distant recurrence	4.702	1.715–12.887	0.003
Therapeutic modality			
Surgery and adjuvant therapy			
Nonsurgical therapy	4.403	0.996–19.467	0.051

*CI* confidence interval, *HR* hazard ratio

## Discussion

In the present study, we found that Masaoka stage and WHO histological classification were the primary determinants of patients’ outcomes and that these factors effectively predicted DFS and OS of patients who underwent complete resection of primary thymoma. Postoperative radiotherapy reduced recurrence rate and was significantly associated with DFS, although it did not serve as an independent predictor. The recurrence pattern of thymoma was an independent predictor of PRS. Although not statistically significant, the type of therapy may be a prognostic factor for patients with recurrent thymoma.

The Masaoka staging system was proposed in 1981 [10], and a modified version was suggested in 1994 [11]. Although the WHO TNM staging system was subsequently proposed, it is not widely used to stage thymoma because of the low frequency of lymph node metastasis [12]. The difference between the Masaoka and WHO TNM staging systems is the classification of N1 disease, which is included into the criteria of stage IVb disease in the Masaoka staging system and stage III disease in the WHO TNM staging system, respectively [13]. Furthermore, the lymph node metastasis rate in patients with thymoma is <2.0%, and the TNM system may therefore be unsuitable for staging thymoma [14]. Similarly, lymph node metastasis was not detected in the thymoma patients studied in the present study. By contrast, the Masaoka staging system, which is most commonly used, focuses on the primary tumor and classifies lymph node metastasis as stage IVb disease. We found that the Masaoka stage served as a perfect predictor of prognosis in the present study, which is consistent with the findings of other studies [3, 15, 16].

The WHO histological classification was first recommended in 1999, and a modification was introduced in 2004. According to the morphology of epithelial cells and the lymphocyte-to-epithelial cell ratio, thymoma is pathologically stratified into types A, AB, B1, B2, and B3 [13]. Interestingly, the prognostic value of WHO histological classification for thymoma is inconsistent in previous studies. Some studies found that the WHO histological classification is an excellent prognostic factor for thymoma and helps clinicians to stratify patients and therefore administer the most appropriate treatment [16, 17]. However, other studies found that the WHO histological classification does not predict DFS and OS [3, 18]. Our results demonstrate that the WHO histological classification was significantly associated with recurrence rate and predicted the DFS and OS of patients with thymoma.

Surgery plays a vital role in the management of thymoma. Compared with thymoma patients who receive conservative therapies, those who undergo complete resection achieve the maximum survival advantage [19]. New and novel surgical techniques such as VATS and the da Vinci Surgical System are now used in clinic. Compared with open surgery, VATS thymectomy shortens postoperative hospitalization, reduces blood loss, and reduces restriction of patients’ activities. These advantages hasten recovery, which was demonstrated by our short-term study [9]. Furthermore, a recent meta-analysis showed that, compared with patients who underwent open surgery, those who underwent minimally invasive surgery (VATS and da Vinci robotic surgery) had less blood loss and shorter hospitalization [20]. For selected thymoma patients, minimally invasive surgery is safe and can achieve survival and recurrence rates comparable with those of open surgery [20]. However, the present long-term study shows that surgical approaches were not significantly associated with survival of patients with thymoma and that DFS and OS did not differ significantly between patients who underwent VATS and those who underwent open surgery (sternotomy and thoracotomy), which may be due to the small size of the patients with recurrent thymoma.

Although complete resection is recognized as curative treatment for patients with resectable thymoma, the role and benefit of postoperative radiotherapy are controversial. The patients treated with surgical resection alone and those treated with postoperative radiotherapy showed significant, though conflicting, results [21], which may be due to heterogeneous subject populations and therapeutic regimens. Our results suggest that postoperative radiotherapy reduced the recurrence rate and prolonged DFS after complete resection of thymoma. We hypothesize, therefore, that patients eligible for postoperative radiotherapy were selected according to the surgeon’s judgment of high risk factors such as invasiveness or intraoperative adhesiveness as well as Masaoka stage and WHO histological classification. Based on our present results and clinical experience, we propose that type B2 or type B3 encapsulated thymoma and invasive thymoma should be treated with adjuvant radiotherapy after complete resection.

Because of the indolent biological behavior of thymoma, long-term follow-up is required for patients who undergo resection. The most frequent site of recurrence (≤92.0%) is the pleura [22–24], which is consistent with the findings of the present study (40%). Locoregional recurrence is the most common reported recurrence pattern [25, 26], which is also consistent with our present findings. The recurrence site was not associated with survival, although here the recurrence pattern was an independent predictor of PRS.

Due to the infrequence of thymoma recurrences, the data about recurrent thymoma are limited, and related studies are rare; the optimal management of recurrent disease is inconsistent and not identified. Surgical resection for recurrent thymoma is recommended because of its feasibility and low postoperative morbidity [27]. In the present study, patients with recurrent thymoma who underwent surgery had a longer PRS compared with those treated with other strategies. Patients who did not undergo surgery had disseminated disease; therefore, the significant differences in survival after recurrence might be explained by disseminated disease that was more extensive. Although the multivariate analysis results of survival after recurrence were not statistically significant, treatment modalities trended as independent prognostic factors of recurrent thymoma (*P* = 0.051). Moreover, prolonged PRS of patients treated with surgery suggests that debulking surgery may play a positive role in the management of recurrent thymoma. To our knowledge, no data are available on the benefit of adjuvant treatment of recurrence. Nevertheless, we recommend the administration of adjuvant therapy to patients with recurrent thymoma after resection because the surgeons cannot intraoperatively find the residual lesions or microscopically recurrent sites.

This study has limitations because of its retrospective nature and the experience of a single center. In a retrospective study, the follow-up schedule could not be made uniform, which may cause bias when assessing the survival time. Even if substantial number of patients underwent complete resection, a randomized large-scale trial is required to further validate our conclusions. Because of the primary selection bias in treatment allocation, it may not be possible to precisely define the role of adjuvant therapy. Furthermore, patients with recurrent thymoma who were eligible for surgery might have survived longer compared with those who were not because of limited dissemination of the tumor.

## Conclusions

In the present study, our results reiterate that Masaoka stage and WHO histological classification are reliable predictors of DFS and OS of patients with thymoma who undergo initial complete resection. The recurrence pattern is an independent predictor of PRS in patients with recurrence. Repeated surgical resection is recommended for patients with recurrent thymoma, along with the possible use of adjuvant therapy.
